# Revisiting delusions to demystify human agency: A response to critics

**DOI:** 10.33735/phimisci.2024.11744

**Published:** 2024-10-14

**Authors:** Lisa Bortolotti

**Affiliations:** a University of Birmingham

**Keywords:** Agency, Authenticity, Comforting delusions, Control, Delusions, Demonising narratives, Epistemic justice, Mental health, Propagandistic cognition

## Abstract

In this paper, I re-elaborate some of the ideas presented in *Why Delusions Matter* (Bloomsbury 2023) in response to four commentaries on the book. My proposed conception of delusionality cuts across clinical and non-clinical contexts: an interpreter calls a speaker’s belief *delusional* when the belief seems to be central to the speaker’s identity, but the interpreter finds it both implausible and unshakeable. Here I frame the emphasis on what all delusional beliefs have in common as an attempt to resist simplistic dichotomies about human agency. *Ideal* agents seek the truth and accurately represent the world, engaging with it in such a way as to further their goals, exercise control over themselves and the surrounding environment, and successfully coordinate with others. *Human* agents can sometimes rely on comforting illusions and constructed worlds to establish or restore a connection with other agents and the shared environment at challenging times. But coordination can fail, and control is always limited. Delusions are an expression of agency in the sense that they can be understood as a response to the epistemic and psychological needs of human agents. Delusions are meaningful and may contain germs of truth about the speaker. But they should be replaced when better strategies for engaging with the world become available to the speaker.

## 1 Two strategies to tackle delusionality

*Why Delusions Matter* (WDM) is a book about the circumstances in which our beliefs are called delusional. It is also a broader reflection on the strengths and limitations of our agency and on our interactions with agents who have different perspectives on the world.

In lay and philosophical conceptions of delusions alike, delusions exemplify the more problematic aspects of agency. We should seek the truth; delusions offer comfort instead. We should be true to ourselves; delusions are not who we really are but an expression of our illness. We should exercise control over ourselves and others; delusions are a sign that things are getting out of control. We should pay attention and assign credibility to those agents who tell the truth, consistently and reliably; people who report delusions demand attention and credibility but fail to be reliable testifiers in the light of their interpreters.

One strategy in WDM and other empirically informed work on delusions (Gunn & Larkin, 2020; Lancellotta, 2022; Ritunnano et al., 2022; Ritunnano & Bortolotti, 2022; Sullivan-Bissett, 2020) is that we should argue for a more balanced role for delusions in our cognition and agency. Delusional beliefs are not always false and are not always a manifestation of illness. People who report delusions need not be out of control and there is no reason to challenge their role as reliable testifiers in contexts other than the delusional context. Even within the delusional context, a person’s beliefs are meaningful and may contribute to a better understanding of the person’s place within the shared environment. As we all have some delusional beliefs in the WDM broad conception of delusionality, this strategy has wide implications for our interpersonal relationships. I call this strategy the *redemption strategy* and I will consider it further in section 3.

But in the following section, section 2, I will focus on another strategy, the *anti-idealisation* strategy. The main point is that several analyses of delusionality, including those that deny a positive role to delusions and those that aim at fully restoring credibility for delusional beliefs, derive from an idealised conception of human agency where human agents pursue true and rational beliefs for the sake of truth and rationality, and this enables them to exercise control over themselves and their environment. The anti-idealisation strategy that pervades WDM rejects the dichotomies associated with human agency as unrealistic and unhelpful when applied to delusions and to the rest of our cognition.

## 2 The anti-idealisation strategy

Here is a myth about human agency: We only benefit epistemically from the truth. Having an accurate representation of reality is a precondition for our being effective agents. Here is a more realistic conception of human agency: We benefit epistemically from beliefs (whether true or false) that enable us to exercise our agency. Having an accurate representation of reality is not a precondition for our being effective agents.

Here is another myth about human agency: Agency is about being in control and fulfilling our goals via the most appropriate means to our ends. As illness is something that escapes our control, it threatens our agency. Here is a more realistic conception of human agency: Agency is about navigating our limitations in perception, cognition, and affect, and multiple constraints due to our surrounding physical and social environment in order to pursue our goals via the means available to us. As illness is an obstacle to the pursuit of our goals, it brings to the fore the strengths and limitations of our agency.

### 2.1 Should I seek comfort or the truth?

In her generous and insightful commentary, Elly Vintiadis considers an objection often raised against psychedelic-assisted therapy, that it engenders comforting delusions. Answering Michael Pollan, who asked: “Is psychedelic therapy simply foisting a comforting delusion on the sick and dying?”, Vintiadis argues that, if psychedelic-induced mental states are not delusional, then the comforting delusion objection does not rule out psychedelic-assisted therapy. Vintiadis applies the notion of delusion developed in WDM to arrive at her reasoned answer: if delusions are what interpreters ascribe to us when we report identity beliefs that they find implausible and unshakeable, then psychedelic-induced mental states are not delusions.

The new understanding of delusions put forward can also help dispel objections like the Comforting Delusions Objection by showing that beliefs acquired during psychedelic states in the process of PAT are not delusional, properly understood: they do not necessarily satisfy the jointly sufficient criteria of implausibility, unshakability and centrality to identity. Making this clear could, in turn, open the way to a form of therapy that, so far at least, holds a promise to help people that cannot be helped otherwise. (Vintiadis, this issue)

Vintiadis is right that psychedelic-induced states are not likely to fit the requirements of delusionality, and this seems true whether one is persuaded by the WDM account of delusions as identity beliefs that others find implausible and unshakeable, or one prefers the more traditional definition of delusions as false and irrational beliefs. But I guess the comforting delusion objection can survive this terminological and conceptual issues: isn’t it just epistemically bad to induce—in people who are unwell—states that have *epistemic* costs just because such states might bring *psychological* benefits? The objection thrives on the assumption that we cannot have both knowledge and happiness and need to choose between (a) opening our eyes to the harsh reality surrounding us, taking in the evidence, and (b) abandoning ourselves to a world of fancy, that empowers and comforts us but is ultimately a deception.

As WDM hopefully begins to show, this is a false dilemma. Unshakeable and implausible (but also false and irrational) beliefs can have significant epistemic benefits that are often temporary. They may at the same time empower and comfort us and enable us to interact with the surrounding environment in such a way as to facilitate learning and knowledge exchanges. A classic example is that of positive illusions: it is because we believe that we are better singers than we actually are—something that makes us proud and happy—that we enter a national singing competition. By doing so, we are in a better position to appreciate what it is that we can and cannot do with our voice and we might realise that what we perceived as our talent will not be sufficient to win the competition. We need further practice and coaching.

Is it true that we are good singers? No. Would we be easily persuaded by a well-meaning friend that we are mediocre singers? Again, no. We are more likely to believe that our friend’s words are driven by their being envious of our remarkable talent. That is because we selectively update our beliefs to project a positive image of ourselves (Jefferson et al., 2017; Sharot, 2011). But the experience of the competition, listening to many singers that are obviously more talented than we are, and consistently receiving critical feedback by experts, has the potential to teach us something, something we wouldn’t have learnt if we hadn’t entered the competition. An inflated belief about ourselves and our talents has been instrumental to our engaging with our environment in a way that enables us to learn something.

Human agents seek both comfort *and* truth, and at times they obtain truth via comforting beliefs, and comfort via truthful beliefs. The case of psychedelic-assisted therapy seems to me to be precisely one context in which it is misleading to claim that we are giving up on the truth to gain comfort.

### 2.2 Are delusions me or my illness?

Lay conceptions of agency (and often philosophical conceptions of agency as well) have it simple. Either we are in control (we are the puppeteer) or we are being controlled by another (we are the puppets). But for the puppet show to bring a story to life, the puppeteer’s skills are not sufficient. There needs to be the right script, the puppets need to look the right way, there need to be the right props, and the audience needs to respond in the right way to what happens on stage. In other words, when the puppeteer makes the puppets move and speak, they are constrained by a number of factors, including the demands of the script, the appearance of the puppets, the props available, the age of the audience, and the size of the stage. The show is never just the puppeteer’s doing, as a belief we endorse is never just something we have produced ourselves.

What I am suggesting here is that human agency is about navigating a complex system of environmental factors and relationships so we can carve some space for finding meaning and purpose in what happens to us. If we ask whether we are puppets or puppeteers when it comes to delusions, we might miss some important aspects of the experience of having a delusion. We are neither. There is a show—the delusion being the show—and we take some authorship of the show by claiming it as ours, but we are not strictly speaking responsible for every single part of it and, when we do make decisions about what is going to happen, we are working with what is available to us.

When delusions emerge as a protection, they are not typically the product of a deliberation process—not many of our beliefs are. They emerge as potential explanations of things that are difficult for us to understand and cope with, and we latch onto them because they are temporarily and imperfectly useful. This is my picture of how we believe things in general, and it applies to delusions as well. (Better: a careful attention to delusional beliefs helps us realise that this is the case for most of our beliefs).

There is no explanation of how beliefs come about that applies to delusional beliefs only—there is never absolute control or total lack of control, there is always partial control. We are constrained, imperfect agents when we come to believe that there are insects crawling under our skin in something like parasitosis, and we are also constrained, imperfect agents when we try to decide which train to catch to get to the other side of the city. The dichotomy between being fully-in-control and being out-of-control is unhelpful as we are never fully in control or completely out of control. We are always somewhere in between. Obviously, the extent to which we exercise control matters, and that is why the question whether a belief is “me or my illness” makes sense to us.

In a very interesting commentary, Cristiano Bacchi compares my view of delusions as not necessarily dysfunctional and as meaningful manifestations of agency to Justin Garson’s account of delusions as a strategy. There are some well-observed areas of overlap, but my model differs substantially from Garson’s view of madness as Garson’s involves a rejection of the medical model. On my view, beliefs are not the type of thing that can be pathological in isolation from other things—that is because pathology is a state of a person as whole. But, even if there could be pathological beliefs, influential philosophical accounts of disorder in terms of either dysfunction, harm, or a combination of the two do not fit what we know about delusions (Bortolotti, 2022). That is because delusions are not always harmful and there is no obvious cognitive dysfunction responsible for their formation.

Although I don’t find the notion of disorder necessary or illuminating in demarcating the scope of psychiatry (or medicine more generally), I believe medical interventions can be appropriate in attempting to ameliorate various life problems we might have, including those relevant to the endorsement of the beliefs that are commonly described as clinical delusions (Bortolotti, 2020). More to the point, I recognise that delusions can be irrational and harmful, and I think these features often coexist with the sense of meaningfulness and protection that delusions can give us. This tension is not, again, unique to clinical delusions: instances of self-deception can at the same time compromise our self-understanding and support our capacity to interact with other agents and the shared environment.

Madness as a whole, and delusions in particular, may be a strategy we consciously adopt to further our aims, but they don’t need to be. They can be legitimate though unsettling responses to something unexpected, distressful, overwhelming. In the account defended in WDM, the absence of a conscious, deliberate strategy does not make madness the antithesis of agency. When we treat madness as the antithesis of agency we do so because we have in mind the traditional picture of the agent as the puppeteer, where the puppeteer’s power goes unchallenged in a world of disposable puppets. We never have that, whether we are delusional or not.

More to the point, having a strategy is a manifestation of an advanced form of agency but it is not all that agency is. When I talk about delusions being a manifestation of agency, I don’t mean that they are a strategy we consciously and deliberately adopt to overcome the obstacles on our way. And I don’t mean either that they are forced upon us by a malevolent supernatural power or by a dysfunction of some sort. What I mean is that our skills as puppeteers give us some wriggle room to express ourselves although we are not exclusively in charge of, and responsible for, the show the audience witnesses. What this wriggle room is, is something worth investigating further, and it may be that in different circumstances it takes different forms.

Bacchi makes some promising suggestions about how a non-pathologising view of delusions can help us revisit the very objective of therapeutic interventions:

If a condition is perceived as abnormal and disordered, it logically follows that efforts should be made to eradicate it. Appreciating the protective function that delusions may serve can mitigate such pathologising perspectives. I contend that attending to the challenges surrounding ambiguity and authenticity has the potential to revise the objective of therapeutic interventions – eliminating one’s own psychiatric condition may not always be desirable. The task of psychotherapy thus evolves beyond symptom eradication to assisting the patient in their own endeavour: to disentangle, to disambiguate, to pursue authenticity in the midst of ambiguity (Bacchi, this issue).

One of the factors that might give us partial control over ourselves and our situation is whether we are in a position to take a stance with respect to our illness. How we view our condition is an expression of agency that enables us to preserve or restore authenticity.

## 3 The redemption strategy

In previous work I have always been resistant to offering an account of the nature of delusions, although I have argued that they are not epistemically irrational in a way that differs qualitatively from how non-delusional beliefs are epistemically irrational (Bortolotti, 2010). In WDM, more boldly, I propose a perspectival way of understanding delusions as those beliefs that an interpreter attributes to a speaker when the speaker reports an identity belief that the interpreter finds implausible and unshakeable. One of the consequences of this account is that it cuts across clinical and non-clinical instances of delusions.

Although my broadened account of delusionality may address some long-standing problems with standard definitions of delusions (for instance, whether delusions can be true), it also raises many questions. Is it helpful to use the label of delusions to encompass beliefs with different features, such as conspiracy theories and delusions of persecution? I think it is fair to say that this account will be helpful for some purposes and unhelpful for other purposes. And this also seems to be the take of commentators Dan Marshall and Jazmine Russell. Their commentaries are pulling in different directions but what persuaded me to address their concerns in the same section is that they are both interested in the more practical and ethical applications of the view presented in WDM, based on my somewhat clumsy claim that we need to “redeem” delusions.

How should interpreters treat the speakers to whom they ascribe delusional beliefs?

### 3.1 Dismissing or engaging?

One risk of highlighting similarities between problematic beliefs, as I do in WDM, is that it becomes tempting then to treat all delusional beliefs in the same way and miss some much-needed nuances. The key is to take the similarities for what they are, a spotlight on some interesting patterns can be identified in otherwise different phenomena. The patterns I see tell a story of continuity between delusional beliefs in clinical and nonclinical contexts: delusion attribution occurs when people with different identity beliefs interact and that the mental state considered as delusional is something the speaker commits to and finds central to how they see themselves in relation to the world.

If I had chosen to focus on the dissimilarities, in aetiology, motivation, areas of application, and so on, the project would have taken a different shape. But acknowledging the differences makes it even more interesting to observe that we tend to treat the person who doesn’t recognise themselves in the mirror and a conspiracy theorist in similar ways. We tend to dismiss their perspectives on the world and stop engaging with them on the topic of their delusion (and often, beyond).

There are stories of discontinuity to be told. Some delusions are reactions to a traumatic event in a person’s life, other delusions are triggered by a significant event in the public sphere. In some clinical delusions, the belief content that is supposed to bring some kind of relief to the speaker can be a truly horrifying story that just happens to reduce the uncertainty caused by a perceptual anomaly. In the demonising narratives common in non-clinical delusions, the belief content may often start off as a disingenuous attempt to create a scapegoat or push a corrupted and divisive political agenda. And sometimes, because life is complicated, the neat divide between an inescapable and innocuous clinical delusion and a dangerous and insidious conspiracy theory gets blurred again, as difficulties can emerge in telling apart the two when they are presented as tales of persecution.

When reflecting on clinical delusions, conspiracy beliefs, optimistically biased cognitions, and extremist beliefs, there are many significant dissimilarities which I often highlight in WDM. These concern the ways the beliefs are formed, endorsed, maintained, and manifested in our lives. However, I chose to point to a potentially common quality they have, which I call *delusionality*. In characterising delusionality I start from a mere observation of our current folk-psychological practices: we often describe other people’s beliefs as delusional, and this happens even when the people we describe in this way carry no psychiatric diagnosis and display no marks of madness. Due to the beliefs attracting an attribution of delusionality, the views of people endorsing and defending those beliefs are likely to be *dismissed outright*, rendering further engagement, analysis and evaluation unnecessary and inappropriate.

An interpreter’s common reaction to a speaker whose beliefs have been labelled as delusional is *disengagement*. In the latter part of WDM, I argue that we should not dismiss the perspectives of people who are attributed delusional beliefs just because the beliefs have attracted that label. There is a lot that we can learn (often about the speaker but also about the world we share with the speaker) from engaging with the beliefs. If I am right and we should not assume that delusions are pathological beliefs, by default un-understandable and meaningless, their presence should not be on its own sufficient reason to dismiss the speaker’s perspective. Naturally, there may be other reasons why interpreters think that the speaker’s view should not be taken seriously and engaged with, and those reasons deserve independent consideration.

In a characteristically lucid and thought-provoking commentary on WDM, Williams is concerned that only some of the beliefs we call delusional deserve a better reputation than they currently enjoy. In particular, he worries that demonising narratives due to propagandistic cognition, such as spreading beliefs about a person being a witch to cause outrage against them, fit the WDM criteria for delusional beliefs and yet have no redeeming features.

[O]ne might worry about the utility of a concept that bundles together clinical delusions of the sort that arise in conditions such as schizophrenia with forms of epistemic irrationality that bear no causal relationship to pathology of any kind. If the concept of delusions becomes so broad that it picks out beliefs with very different aetiologies and consequences, reliable generalisation becomes challenging. This might be a problem for the revisionary aspect of WDM’s project, which seems to involve such a generalisation: namely, that delusions in general deserve a more positive reputation than they currently enjoy. For example, Bortolotti (Bortolotti, 2023b, p. 12) argues that the “dismissal of the speaker’s perspective [in cases of delusion attribution] is something we can work harder to avoid as interpreters”. I agree that this is important when it comes to clinical delusions. However, it is not obvious that it is always or even typically the most appropriate course of action when it comes to nonclinical delusions. (Williams, this issue)

Are demonising narratives delusional beliefs as I characterise them in WDM? Are they a legitimate counterexample to the view that we should not dismiss the speaker’s perspective? Demonising narratives are (often stereotypical) examples of delusional beliefs. First, they are often something the speaker is genuinely committed to. In some cases, as Williams argues, the speaker comes to believe the demonising narrative even if it is something they initially concocted for self-serving purposes. So, demonising narratives meet the belief criterion.

Are demonising narratives implausible and unshakeable? Many interpreters find demonising narratives implausible and lacking empirical support, and speakers are unlikely to give them up in the face of objections, given that they need them to justify their own behaviour and they are likely to base on them further beliefs and plans of action. So, it would seem that demonising narratives meet the unshakeability and the implausibility criteria.

Are demonising narratives identity beliefs? Williams offers a very interesting discussion of this point, with careful considerations pro and against, and the conclusion I draw from it is that we should be open to the possibility that at least some demonising narratives are central to the identity of the people who spread them and believe them. Speakers attempting to undermine someone’s credibility by spreading the rumour that the target person is a witch may find this strategy successful and adopt it to the extent that they consider themselves as “witch hunters”, first and foremost, and characterise their contribution to society in those terms. Some people dedicated their lives to hunting witches and saw that as a mission, as something that gave them value and purpose in the eyes of others. So, the identity belief criterion can be met too.

Some demonising narratives meet the WDM criteria for delusionality. Do they have the “redeeming features” I attribute to delusions? I don’t see why not. After all, what I argue in WDM is that delusions (1) are not pathological beliefs; (2) are meaningful to speakers; (3) can have psychological and epistemic benefits as well as psychological and epistemic harms for speakers (whilst having harms for society more widely); (4) cause speakers to be treated as someone whose perspective should be dismissed outright.

Demonising narratives are not very plausibly pathological; indeed, Williams describes them as an adaptive strategy to decrease the social status of the target or eliminate people who are seen as a burden or a danger for the community. Demonising narratives are meaningful to the speaker; as Williams say, speakers may fabricate them to start with, but often end up genuinely believing them and interpreting important events in their lives using such narratives. That is because speakers are drawn to develop numerous arguments for the demonising narratives in order to persuade other people to embrace such narratives and those arguments end up persuading themselves as well. Beliefs that can be argued for and become central to a speaker’s identity are most certainly meaningful and they can also give meaning to the speaker’s other beliefs and behaviour, reinforcing their ideological system—whether such system is something we value or condemn. Demonising narratives are most certainly psychologically and epistemically beneficial for speakers; as Williams says, they legitimise other beliefs that people have, reducing dissonance; they present the speaker and their group as superior to other members of the community and to rival groups, including the target group, making the speaker feel powerful and in control. Maybe demonising narratives do not emerge to satisfy the speaker’s epistemic and psychological needs. But they are certainly well placed to satisfy some of those needs once they are in place, including the need to find a villain to whom the responsibility for previous calamities and personal failures can be conveniently attributed.

I guess the very real challenge is as follows: why should we engage with speakers who spread demonising narratives, which are harmful to the targeted individuals and society as a whole? Isn’t propagandistic cognition the perfect example of a case where dismissing a speaker’s view is the appropriate thing to do to contain the damage that their view can cause? The short answer is to say that we should not dismiss the speaker’s perspective due to their being ascribed delusional beliefs. But we may have other reasons to dismiss the speaker’s perspective and some of these reasons may be good reasons. For instance, if we have reason to believe that the speaker’s perspective, once voiced and engaged with, will cause serious harm to vulnerable people, then it may be justified to avoid engagement with the speaker.

An interesting point to consider is whether dismissing people endorsing demonising narratives is the right thing to do if we care about the long-term consequences of our mutual interactions for the public debate. It is not clear that dismissing the authors of demonising narratives will ensure that they cause as little damage as possible. It may be an effective short-term containment strategy. But often when proponents of demonising narratives are denied a platform, being actively ignored and excluded from debate, they present themselves as victims and gain the sympathy of others, managing to turn their self-serving, harmful beliefs into cries for freedom and independent thinking. Careful engagement could expose demonising narratives for what they are, and help interpreters offer speakers less damaging ways to address their psychological and epistemic needs.

### 3.2 Compassion or justice?

In a beautifully written commentary on WDM, Russell argues that my redemption strategy is not enough. There is a lot to commend in the idea that delusions have significant benefits for speakers and that delusions are responses to speakers’ psychological and epistemic needs. But we should take this further and recognise that delusions are alarm bells, revealing something important, often urgent, about ourselves and our bodies that needs to be addressed.

Bortolotti also rejects the idea that delusional beliefs themselves are pathological, even if some biological pathology may be present (2023a). However, she maintains that delusions are meaningful yet suboptimal responses to crises. The ‘imperfect cope’ explanation of why delusions are meaningful, doesn’t validate the potential epistemic value within delusions as sometimes containing partial-truths, and instead risks ignoring legitimate concerns. Rather than an imperfect coping strategy to dealing with crisis or challenge, some delusions are meaningful because of their adaptive potential to help alert us to or offer information about pathology and harm as they are experienced in the body. Delusions may be a survival strategy when all else has failed. When the body runs out of ways to express its pain, delusions just may be the most functional and reliable way to alert us to pain. (Russell, this issue)

I find this approach extremely interesting, and in my own work I often hint at how delusions offer information about speakers that is true and needs to be addressed. In WDM as well as in previous work (Gunn & Bortolotti, 2018; Ritunnano & Bortolotti, 2022), I consider the truths often hidden in beliefs considered as delusional and I discuss real-life cases suggesting that what the person was saying had a justification in their experience even if it was not something the interpreter could understand or fully appreciate at that time. That is part of the reason why I resist the description of delusions as un-understandable: judgements of un-understandability tell us more about the interpreter’s lack of background information and imagination than about the speaker’s sanity.

Russell objects to the characterisation of delusions as coping mechanisms, but I think our views converge more than is apparent, because what I mean when I call delusions “imperfect responses to crises” is that there are epistemic, not only psychological, needs that the delusional belief responds to, at a time when no other response may be possible. The response is beneficial and valuable in giving us an explanation or strategy that enables to manage the crisis, and yet it may be replaced with another (non-delusional) explanation or strategy when the time is right—that is, when we acquire additional information or we get the resources to process the information we already have that is for some reason hidden from us (and others) or difficult for us (and others) to use.

The reason why I consider the possibility of the delusion being replaced in some circumstances (not in all), is not that the delusion does not meet the potentially parochial standards of plausibility of the interpreter, but that the delusion can cause as well as alleviate harm when it offers information about the speaker in the way it does. While relieving lack of knowledge, uncertainty, and anxiety, and pointing to solutions to existing problems, the delusion can also be a cause of stress, isolation, and harm in its own right, and in such cases it becomes important to be open to seeking an alternative explanation or strategy in the long term. This is again something that applies to human cognition and agency in general. We identify ways of navigating the world that make sense to us and enable us to pursue some of the things that matter to us, and replace them with ways that serve our purposes better when we can.

As mine is a view of continuity, where engagement should not be withdrawn when a person expresses a view that we do not share and find “delusional”, I am surprised when Russell interprets my reflections on what delusions can teach us about mutual interactions as an invite to be compassionate rather than just:

Many attempts at understanding delusions, even with good intention, can become paternalistic. This can be the case whether we view delusions as a sign of a broken brain or cognitive defect, as a mind obscuring itself from a hidden truth, or as a suboptimal way to cope with uncertainty. These are all ways that the listener attempts to understand, but often with an underlying motive of trying to get the speaker to change, either to see their self-deception, faulty logic or to realize the underlying ‘disorder’. These explanations maintain that there are no instances in which delusions are functional and appropriate responses, and are often filled with pity for the speaker, rather than real empathy or respect. Taking the step towards viewing delusions as an adaptive coping strategy to challenging events rather than a pathology is a positive one. However, to “create an epistemic environment where delusions do not constitute an obstacle to mutual understanding” (Bortolotti, 2023a, p. 147) it’s crucial to understand delusions from a lens of epistemic justice, or compassion remains an empty intention (Russell, this issue).

I consistently suggest in WDM that when it comes to delusions we are all sometimes interpreters and sometimes speakers, as we all have some belief that others find implausible and unshakeable and that defines us. I never use the language or conceptual framework of compassion in WDM, and I dedicate the last chapter of the book to identify and find amelioration strategies for those acts of injustice that occur when the ascription of a delusional belief causes the interpreter to downgrade the speaker’s agency—as I put it there, when interpreters abandon the agential stance towards speakers. The result of the downgrading is disengagement.

Effective solutions to injustices of an epistemic sort cannot be achieved solely at the individual level, because there are systemic issues that need to be addressed, involving the culture of our institutions as well as our good skills and virtues as individual agents. As a partial solution to situations where interpreters dismiss outright those speakers who have been ascribed delusional beliefs, I consider the epistemic virtues of curiosity and empathy, as recommended by the researchers with lived experience who worked with us on a collaborative project, analysing successful and unsuccessful clinical encounters between practitioners and young people accessing mental health emergency in the UK (Bergen et al., 2023). Lived experience researchers on the project observed that genuine curiosity was missing in the practitioners who dismissed the young people’s perspectives during the encounters. There was no attempt to understand what the young person’s experience was and what made it distressing. Often, this led to de-legitimising the young person’s attempt to seek help, suggesting that they did not need to access services after all, or sending them away with no further support. In more successful interactions, practitioners took time to ask questions about the young person’s life and context, trying to understand their experiences and feelings, and what could help them overcome the crisis. Young people were also actively involved in decision making processes (Bergen et al., 2022).

Although the young people whose encounters we analysed for this project did mostly experience problems with their mood, self-harm, and suicidal thoughts, more recent preliminary work with the Voice Collective, whose members are young people with unusual beliefs and experiences, confirmed that the presence of beliefs considered by others as delusional causes people to lose credibility. This leads them to being perceived as dangerous, childish, and lacking capacities and competencies, within but also beyond the domain of their unusual experiences and beliefs. In the narrower context of our discussion, this means that concerns raised by people with a medical history of psychosis, about diagnosis, medication, treatment options, and other issues, are routinely considered as a product of their illness and thus not taken seriously.

Although the last chapter of WDM already briefly addresses the need to adopt an agential stance towards people with (even radically) different perspectives, in clinical and non-clinical contexts alike, new work will offer a more detailed analysis of the types of injustice involved in the mental health context, and make suggestions about promising strategies for amelioration (Bortolotti, 2024).

## 4 Conclusions

I am extremely grateful to the four commentators for their patience in reading the book and for their precious insights, at times highlighting problems and inconsistencies, at other times suggesting some new exciting developments of my view. Their contributions are a great source of inspiration for future work.
